# Do education and living standard matter in breaking barriers to healthcare access among women in Bangladesh?

**DOI:** 10.1186/s12889-023-16346-8

**Published:** 2023-07-26

**Authors:** Kanchan Kumar Sen, Shahnaz Nilima, Fatima-Tuz Zahura, Wasimul Bari

**Affiliations:** Department of Statistics, University of Dhaka, Dhaka, 1000 Bangladesh

**Keywords:** Healthcare Access Barriers, Education, Living Standard, Dominance analysis, BDHS

## Abstract

**Background:**

Barriers to healthcare access for women have a substantial influence on maternal and child health. By removing barriers to accessing healthcare, several sustainable development goals can be achieved. The goal of this study, based on the dominance analysis, was to examine how living standards and spousal education play role in removing barriers to healthcare access for women in Bangladesh.

**Methods:**

The study used the nationally representative Bangladesh Demographic and Health Survey (BDHS), 2017-18 data. A binary logistic regression model was applied for analyzing different types of health access barriers in the study. Additionally, a dominance analysis was conducted to identify the most responsible factors for removing barriers.

**Results:**

In Bangladesh, 66% of women faced at least one barrier in accessing healthcare. The results obtained from logistic regression and dominance analysis revealed that women’s standard of living and spousal education explained the highest variation of having at least one barrier in accessing healthcare. Specifically, a high standard of living explained 24% of the total explained variation (OR 0.56, 95% CI 0.52–0.62), while both spousal education accounted for 27% (OR 0.49, 95% CI 0.45–0.54) of the total explained variation. The regression results also showed that women with higher standards of living as well as educated women having educated partners had lower odds of facing barriers in getting permission (OR 0.87, 95% CI 0.76-1.00 and OR 0.66, 95% CI 0.58–0.75) to go for advice/treatment, obtaining money (OR 0.43, 95% CI 0.39–0.47 and OR 0.37, 95% CI 0.34–0.40), distance to a health facility (OR 0.60, 95% CI 0.55–0.66 and OR 0.70, 95% CI 0.65–0.76), and not wanting to go alone (OR 0.72, 95% CI 0.66–0.89 and OR 0.75, 95% CI 0.69–0.81) for getting medical advice/treatment.

**Conclusion:**

The findings of the study suggest paying extra attention to the spousal education and living standard of women to strengthen and reform the existing strategies and develop beneficial interventions to enhance unhindered accessibility to healthcare facilities for women.

**Supplementary Information:**

The online version contains supplementary material available at 10.1186/s12889-023-16346-8.

## Background

Healthcare access may have significant impact on health outcome [1, 2]. Access to quality healthcare necessarily contributes to promote and maintain good health, prevent, and treat diseases, reduce the risk of infirmity and premature deaths, and achieve equity in health [3, 4]. Ensuring affordable and better-quality healthcare to everyone is the main focus of universal health coverage (UHC) in order to meet third sustainable development goals (SDG) targets. Although many countries have made progress towards achieving UHC, vulnerable groups especially women and children from developing countries still face healthcare access barriers [5].

Women’s role is important for socioeconomic development of every society, and their health is prioritized as a key public health concern worldwide [6, 7]. The SDGs 3.7 and 3.8 emphasized on women’s good health and well-being. Despite some progress towards these targets, about 295,000 women die yearly worldwide due to pregnancy and childbirth related complications [8]. Although a large proportion of women die globally due to non-communicable diseases such as cardiovascular diseases, cancers, diabetes and chronic respiratory diseases, prevalence of these diseases is comparatively higher in developing countries [9, 10]. However, utilization of accessible and affordable better quality health care services could avert women’s morbidity and mortality.

Antenatal care (ANC), delivery care (DC) and postnatal care (PNC) are important for better maternal and neonatal health outcomes [11]. Barriers in accessing these maternal health care services among women may lead to adverse health outcomes like pregnancy complications, miscarriage, stillbirths [3, 10, 12]. Poor child health outcomes are negatively related with utilization of maternal health care services [13]. Moreover, ANC and skilled birth attendance could address major causes of maternal and neonatal deaths [14]. Therefore, access to these health care services could reduce maternal as well as child mortality [15, 16]. Again, women’s access to family planning services plays an important role in improving maternal and child health by preventing sexually transmitted diseases, unintended pregnancies, and unsafe abortions [11, 17, 18]. Therefore, ensuring access to maternal and reproductive health care services for women is a prerequisite for reducing maternal and child mortality.

Bangladesh is one of the developing countries moving towards UHC. Despite some achievements have been made since 2000, health care utilization indicators for women (family planning demand satisfied- 70%, at least four antenatal care visits- 47%, institutional delivery- 49%, skilled birth attendance- 53% and postnatal care- 52%) are low [5, 11]. Several factors could prevent women from accessing health care services. These barriers could include financial problems, geographical location, transportation, health literacy, health infrastructure and psychological barriers [10, 19]. Previous studies revealed that distance and money for treatment were major barriers perceived by women in accessing health care [3, 10]. However, lack of access to health care services increases the risk of adverse health outcomes [20]. Although Bangladesh has made progress in reducing maternal and child mortality, the targets of SDG 3 are still unmet. To achieve these targets, Bangladesh needs to address the factors responsible for women’s barriers to healthcare in order to ensure women’s access to quality healthcare.

Access to healthcare for women is a big challenge in Bangladesh, particularly in rural areas where poverty, lack of education, and cultural norms can prevent women from seeking or receiving adequate healthcare services [21]. There are several factors that can contribute to these challenges, including limited availability of healthcare facilities and services, insufficient numbers of healthcare professionals, and inadequate health education and promotion programs [22]. Additionally, there are cultural barriers that may prevent women from seeking healthcare, such as social stigma around certain health conditions, lack of female healthcare providers, and gender-based discrimination. To address these challenges, the government of Bangladesh has implemented several initiatives to improve access to healthcare for women. For example, the government has established community-based healthcare programs that provide basic healthcare services to underserved populations, including women [23]. The government has also increased investment in the healthcare sector, which has led to improvements in infrastructure and human resources. Non-governmental organizations (NGOs) have also played an important role in improving access to healthcare for women in Bangladesh. NGOs such as BRAC and CARE have implemented programs that focus on maternal and child health, family planning, and nutrition [24]. These programs provide health education and promote behavior change to improve health outcomes. Despite these efforts, challenges to accessing healthcare for women in Bangladesh persist. According to a recent health survey report in Bangladesh, 67% of women aged 15–49 faced barrier to access health care services [11]. Therefore, the present study focuses on women’s standard of living and education as key factors that can play a significant role in accessing healthcare among women in Bangladesh.

Education is considered as a basic social determinant of health [25]. Globally, access to healthcare or health outcomes are significantly influenced by the education of women and their partners [6]. Women with more education are better informed about nutrition and health, and they are more likely to seek medical attention when necessary. Also, they are more likely to take responsibility for the health of their family and make wise healthcare decisions. Partner education can also play a role in improving healthcare outcomes by creating a supportive environment for healthcare-seeking behavior. Earlier studies reveal that better education of women and their husband help to understand the usefulness of ANC, DC and PNC services, and hence result in more utilization of these services [15, 16]. Moreover, evidence suggests that low education level obstructs women to access health care services. Furthermore, education also reduces gender inequalities and assists women to be empowered [26]. Educated women are more likely to be conscious of their rights and to advocate for them. The cultural norms and beliefs that support gender disparity can also be overcome with education [27]. In addition, educated women tend to have higher incomes and are more likely to participate in the labor force which can lead to break the barriers in accessing healthcare.

Health outcomes are also dependent on standard of living of a household. Generally, people with higher standards of living tend to have better health outcomes than those with lower standards of living [28]. This may be because those with higher standards of living have better access to healthcare, better nutrition, and safer living conditions. According to earlier research, people with poor quality of living are more likely to experience chronic stress [29], which can lead to several health problems like diabetes, cardiovascular disease, and mental health disorders, all of which can make it more difficult to access healthcare. In addition, persons with poorer standards of living may have less access to healthcare due to financial constraints, which makes it challenging to treat pre-existing conditions or get preventive care. In order to increase women’s access to healthcare, improving the standard of living can be a key strategy. Several studies have been conducted to determine the factors associated with barriers to health care among women of reproductive age. Literature found that women’s empowerment, women’s education, age, husband’s education, wealth index, religion, place of residence, number of living children have significant impact on women’s accessibility to health care [4, 5, 12, 20]. To our knowledge, there is dearth of studies that assessed the barriers that women face to access health care in Bangladesh. Therefore, understanding the reasons of health care access problems among women are crucial for better maternal and child health outcomes in Bangladesh. In the present study, the spousal education and living standard are considered as important predictors of barriers to healthcare access among women, where living standard is a latent phenomenon derived from some household facilities such as electricity access, modern cooking fuels, improved sanitation facility, improved drinking water, improved housing materials. and household assets following the multidimensional poverty index [30, 31].

It is noteworthy that most previous studies have primarily focused on investigating the determinants of healthcare access barriers, rather than specifically examining the impact of factors such as spousal education and standard of living. Our study aimed to contribute to the existing literature by highlighting the importance of both spousal education and standard of living in removing barriers to healthcare access for women in Bangladesh. These factors have not been extensively explored in previous research studies. Furthermore, through our statistical dominance analysis, we were able to identify spousal education and standard of living as the most influential factors. Therefore, this is the first study to specifically focus on the living standards of women and spousal education as important contributors to breaking barriers to healthcare access among women in Bangladesh. Consequently, we selected reproductive married women from a nationally representative cross-sectional survey dataset to serve the purpose of our study. The findings of our study may assist policymakers in designing effective policies, programs, and interventions to ensure better quality and equitable healthcare for women.

## Methods

### Study design

**Fig. 1:**
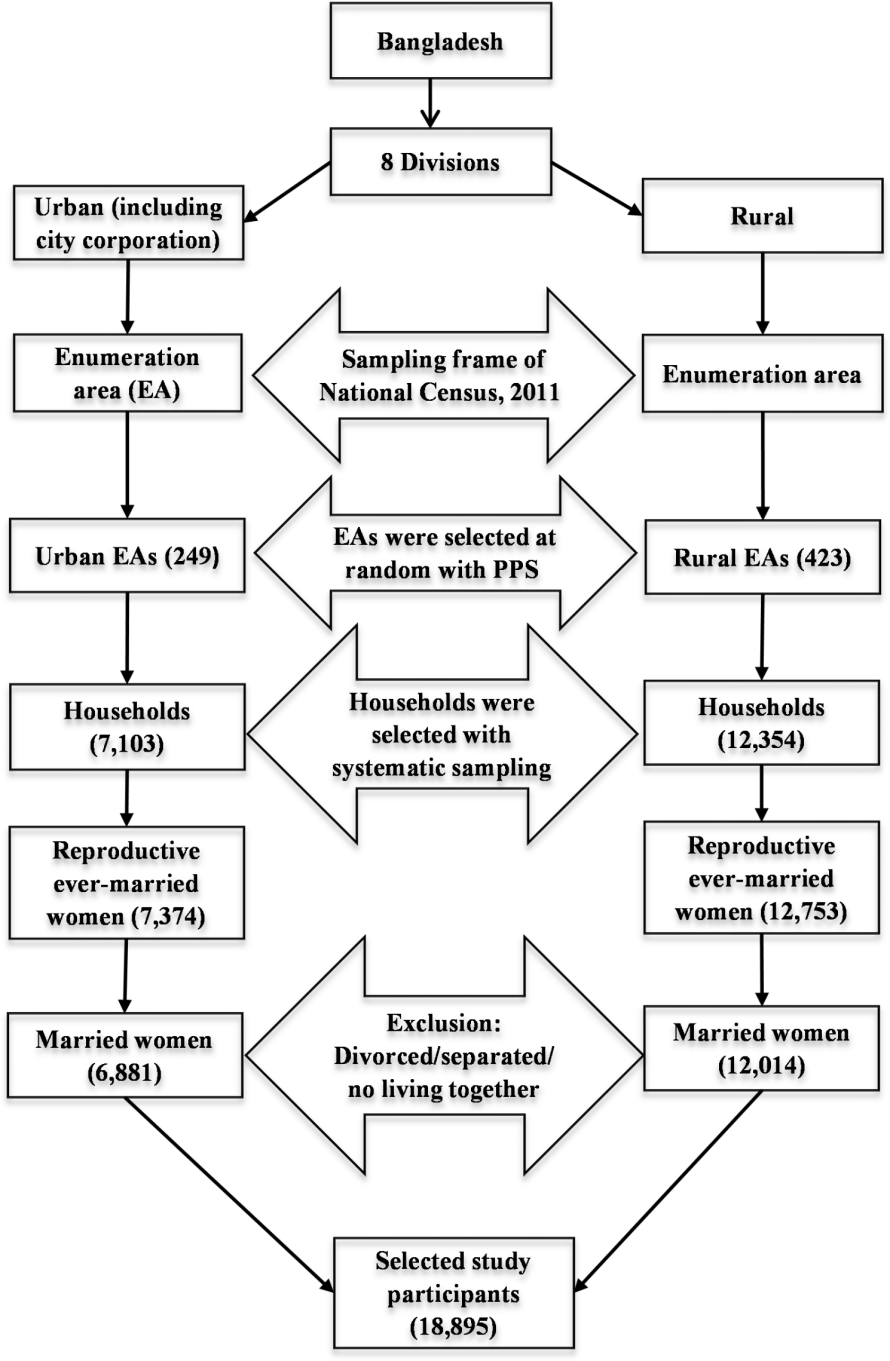
Flowchart for selection of study participants

The study area of the present research was in Bangladesh, and a nationally representative cross-sectional data extracted from Bangladesh Demographic and Health Survey (BDHS), 2017-18 was used to conduct the study. The survey used the sampling frame of the 2011 national census prepared by Bangladesh Bureau of Statistics. The Bangladesh was divided into 22 sampling strata covering the country as whole in the survey. Each stratum consists of a number of enumeration areas (EA) comprised of a full list of households. The data were collected with random following two-stage stratified sample design. In the first stage, a certain number of EAs (675) was chosen from all the EAs with probability proportional to size (PPS) covering each stratum and an average of 30 households were selected from each selected EA considering a systematic random sampling. The survey interviewed all ever-married reproductive women who were either usual members of the selected households or who had spent the night before the survey in the households. However, a total of 20,127 women were successfully interviewed in the survey. Details of the sample design can be found in the BDHS, 2017-18 final report [11]. The women who were either divorced, separated, or not living together with their husband/partner were excluded from the present study, because some selected variables of these women are missing in the BDHS, 2017-18 dataset. Therefore, this study included a total of 18,895 reproductive married women to serve the study purpose. To see the sample selection procedure at a glance, the following flowchart has been constructed (Fig. 1).

### Outcome measures

Access to healthcare is a fundamental human right that is essential for maintaining good health and well-being for women. For many women in Bangladesh, access to healthcare remains a significant challenge due to various barriers. Therefore, the study focuses on some healthcare accessing barriers to identify some potential factors for contributing to breaking the barriers. Following literature [3, 10, 20] and availability of information in BDHS, 2017-18, four types of barriers faced by women in accessing health care were considered which are the outcome measures of this study. These barriers were measured from four questions which were asked women in the interview of BDHS, 2017-18 to know the problem in healthcare access. The questions were on difficulty in getting permission to go for treatment, obtaining money for treatment, distance to health facility and not wanting to go alone (companionship) (Table 1). There were two responses in the questions: big problem and not a big problem, and the questions were transformed to binary variables indicating 1 for big problem and 0 for not a big problem. Each binary variable was considered as outcome variable in the study. Again, the combined of the indicator variables was also considered as dependent variable in the study. For creating the variable, firstly, the indicators were summed to get a score of 0, 1, 2, 3 and 4 (Cronbach’s alpha 0.63), where 0 indicated that women faced no barrier and 4 indicated that women faced all barriers, and then the variable was made as binary measure defining the categories as “yes” if a woman faced at least one or more of the problems (coded as “1”), and “no” if she did not face any difficulty in the problems [3, 10, 20].

**Table 1:** Definition and measurements of the selected variables used in the study

Variables	Definition and Measurement
***Outcome Measures***	For seeking medical advice or treatment for women when they are sick, they were asked whether they faced a big problem for
Getting permission to go for treatment	getting permission to go for treatment (Yes/No).
Getting money for treatment	getting money for treatment (Yes/No).
Distance to health facility	distance to health facility (Yes/No).
Not wanting to go alone	not wanting to go alone (Yes/No).
***Covariates***	
Living Standard	Following a UNDP report, we have created multidimensional living standard index after considering six dimensions named lighting, cooking, household sanitation facility, drinking water facility, housing and household assets, and then categorized the index into three categories-low, moderate and high.
Women’s current age	The women’s current age (in years) has been categorized into four categories- 15–20, 20–30, 30–40, and 40–49.
Early marriage	Women’s early marriage is defined as a marriage before the age of 18. Categories: Yes/No
Women’s education level	In the BDHS, 2017-18 dataset, there were four levels of education: no education, primary, secondary and higher education.
Husband’s education level	The husband’s level of education was also categorized into four categories in the BDHS, 2017-18 dataset: no education, primary, secondary and higher education.
Empowerment status	Women’s empowerment has been assessed using the variables regarding their participation in household decisions. In the DHS, women were asked who decide on (i) spending her partner’s earning, (ii) her own health care, (iii) large household purchases, and (iv) visits to family relatives. In the study, the four binary variables have been created, where a value of 1 is given for those women who take decisions alone or jointly with her husband in the above cases, and 0 for otherwise. Finally, the binary variables are summed to get a score of 0, 1, 2, 3 or 4. These scores are grouped as- non-empowered (0), partially empowered (1, 2 or 3), and fully empowered (4).
Attitude towards intimate partner violence	This variable is measured based on the perception towards wife beating. It is measured based on five indicators. Women were asked if they agree that a husband is justified in heating/beating if she burns the food, she argues with him, she goes out without telling him, she neglects the children, and she refuses to have sex with him. If at least one is true, a woman is treated as violated by her partner/husband.
Employment status	The employment status includes several occupations in the original BDHS dataset, but we re-categorized the employment status into four categories in the study-no paid work, agricultural/domestic services/unskilled manual, sales/skilled manual, and professional/technical/managerial/ services
Religion	The religion has been re-categorized into two categories as Muslim (Islam) and Non-Muslim (Hinduism, Buddhism, and Christianity) in the study.
Number of living children	Women’s number of children has been divided into two groups as below 3 and 3 or more children.
Place of residence	Urban/rural
Region	The whole Bangladesh is divided into three regions based on the divisions: Central (Barisal, Mymensingh and Dhaka divisions), Eastern (Chattogram and Sylhet divisions), and Western (Khulna, Rajshahi and Rangpur divisions) [32].
All-weather road	The name of original variable was main access road which includes all-weather, seasonal, waterway, path, and others in the BDHS. But, in the study we have categorized the variable into two categories as having all-weather road or not.

### Main exposures

#### Living standard

The living standard is a latent variable that was derived from some household facilities. In the study, an index was created based on electricity access, cooking fuels, sanitation facility, drinking water, housing materials and household assets to define the living standard of a household. To measure the index, the Human Development Report developed by the Oxford Poverty and Human Development Initiative (OPHI) with the United Nations Development Program (UNDP) was followed in the study [30]. All the indicators of living standard were transformed to binary variables, denoting 1 for deprived in the respective indicator, to create the deprivation score of living standard. To get the score, the indicators (Cronbach’s alpha 0.72) were summed considering the equal weights for each indicator’ following the UNDP report [30]. Mathematically, the deprivation score in living standard of a women can be written as follows

$$ {y}_{i}={\sum }_{j=1}^{d}{w}_{j}{I}_{ij}$$,

where $$ {y}_{i}$$ indicates the living standard deprivation score of $$ {i}^{th}(i=1, 2, \dots , n)$$ reproductive married women; $$ {w}_{j}$$ denotes the weights in the $$ {j}^{th}$$ dimension (here is $$ \frac{1}{6}$$) so that $$ {\sum }_{j=1}^{d}{w}_{j}=1$$; $$ {I}_{ij}$$ stands for indicator value (either 1 or 0); $$ d$$ and $$ n$$ indicate the number of dimension to measure living standard and number of selected women, respectively [33]. Theoretically, the score lies between 0 and 1, where 0 represents no deprivation and 1 indicates full deprivation in living standard. Details of the dimensions, indicators and weights of living standard have given in Table 2. The study used the cut-off values of UNDP report to categorize the living standard of a household into three categories: low (if deprivation score exceeds 0.5), moderate (if deprivation score was between 0.33 and 0.5) and high (if deprivation score does not exceed the cut-off point 0.33) [30, 31].

**Table 2:** The dimensions, indicators, deprivation cutoffs and weights of household living standard

Dimension	Indicator	Deprived if	Weight
**Lighting**	Electricity Access	The household has no access to electricity.	1/6
**Cooking**	Modern Cooking Fuels	The household has no clean cooking fuels (electricity, natural gas, kerosene or biogas).	1/6
**Sanitation Facility**	Improved Sanitation	The household does not have the followings: improved toilet facility (flush toilet, flush to piped sewer system, flush to septic tank, flush to pit latrine, pit latrine with slab or ventilated improved pit latrine), no sharing the toilet with other households, and improved hand washing materials (liquid/bar soap or detergent)	1/6
**Drinking Water**	Improved Drinking Water	The household does not have the improved source of drinking water (piped water, tube well/borehole, protected dug well, rainwater, tanker or bottled water) or does not treat water to make safer for drinking.	1/6
**Housing**	Improved housing Materials	The household does not have the followings: improved floor (parquet or polished wood, vinyl or asphalt strips, ceramic tiles or cement), improved roof (metal, wood, calamine/ cement fiber, ceramic tiles, cement or roofing shingles) and improved main wall (tin, cement, bricks, cement blocks or shingles).	1/6
**Assets Ownership**	Household Assets	The household does not own more than one water pump, air conditioner, computer, mobile telephone, radio, TV, refrigerator, bike and does not own a car or truck.	1/6
**Total**			1

#### Spousal education

To measure the spousal education, we have re-categorized both husband’s and wife’s education levels. Both levels of education have been converted into two categories: uneducated (no and primary) and educated (secondary and higher). Finally, the variables were combined (Cronbach’s alpha 0.65), and then the spousal education has been categorized into four categories: both uneducated, wife educated only, husband educated only, and both educated.

### Control variables

Following the previous studies, women’s current age, early marriage, empowerment status, attitude towards intimate partner, employment status, religion, number of living children, place of residence, region and all-weather road were considered as control variables in the study [4, 5, 12, 20]. The definition and measurements of the selected variables have been given in Table 2. These control variables were considered in this study to examine how barriers to healthcare access are influenced by spousal education and the standard of living after adjusting the effects of selected control variables.

### Statistical analysis

The data management and analyses were conducted using STATA version 14. The descriptive statistics like frequency percentages of the selected variables and cross-tabulation of all the independent variables against women’s healthcare access barriers were presented with chi-square test in the study. To select the potential covariates in regression analysis, the covariates that were found to be statistically significant at 10% level of significance (p < 0.10) at bivariate were considered to the regression models. For regression analysis, five binary logistic regression models were considered for five response variables (permission to go for treatment, money for treatment, distance to health facility, not wanting to go alone, and at least one barrier in accessing healthcare). The binary logistic regression model using a logit link can be presented as follows

$$ \text{log}\left(\frac{\text{P}\text{r}[Y=1]}{1-\text{P}\text{r}[Y=1]}\right)={\beta }_{0}+{\beta }_{1}SE+{\beta }_{2}LS+\gamma {\prime }X$$

where, $$ \text{P}\text{r}[Y=1]$$ represents the probability of facing barrier in accessing healthcare, $$ {\beta }_{0}$$ is the intercept term, $$ {\beta }_{1}$$ and $$ {\beta }_{2}$$ denote the regression coefficients of spousal education (SE) and living standard (LS), respectively, and $$ \gamma $$ represents set of regression coefficients of other independent or control variables ($$ X$$). The term $$ \frac{\text{P}\text{r}[Y=1]}{1-\text{P}\text{r}[Y=1]}$$ indicates the odds of facing barrier in healthcare access. By taking the ratio of two odds calculated from two groups of women, the odds ratio (OR) can be easily determined.

After identifying the potential factors for breaking barriers in healthcare access using the regression model, the study also tried to explore a most contributing factor. Therefore, to find out the relative importance of the selected predictors in the final regression model (at least one barrier) for reducing women’s barriers in accessing healthcare, the dominance analysis was used in the study [34]. Dominance analysis is a statistical technique that assesses the contribution of each explanatory variable and ranks them according to how important they are for predicting an outcome [35]. In this approach, the independent factors’ individual effects on the outcome variable are considered as well as their combined effects with other independent variables. An explanatory variable’s importance is determined by running a series of regression models (known as sub models) that include all possible combinations of the independent variables, and then McFadden pseudo-$$ {R}^{2}$$ values are calculated for each combination to calculate the general dominance statistic for each predictor [34–36].

## Results

### Prevalence of barriers to healthcare access

**Fig. 2:**
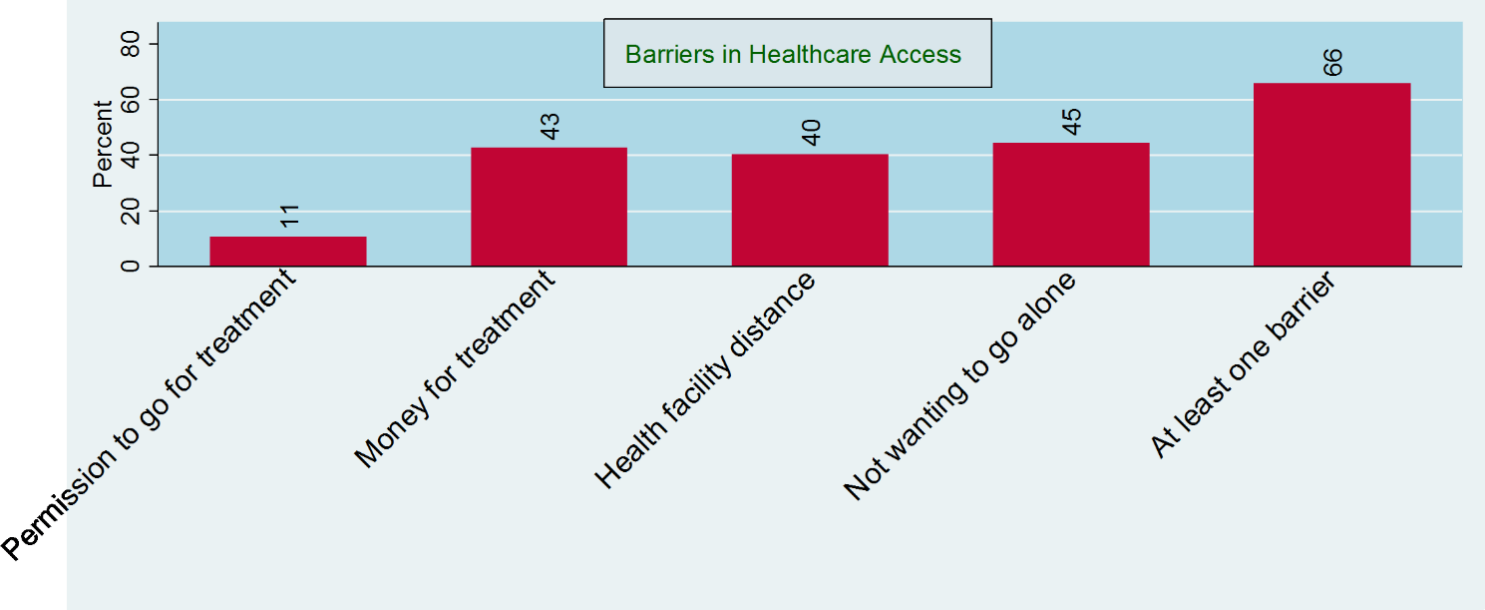
The percentage of reproductive married women having several types of barriers to healthcare access in Bangladesh

Figure 2 shows the percentage or prevalence of facing barriers to accessing healthcare among women in Bangladesh. There has been a low prevalence (11.0%) of barrier in getting permission to go for treatment or seeking medical advice. Other barriers such as money for treatment (43%), distance to health facility (40%) and companionship to go for treatment (45%) occurred almost equally among Bangladeshi women, but about two-thirds of women experienced at least one barrier to accessing healthcare.

### Background characteristics and barriers to healthcare access

**Fig. 3:**
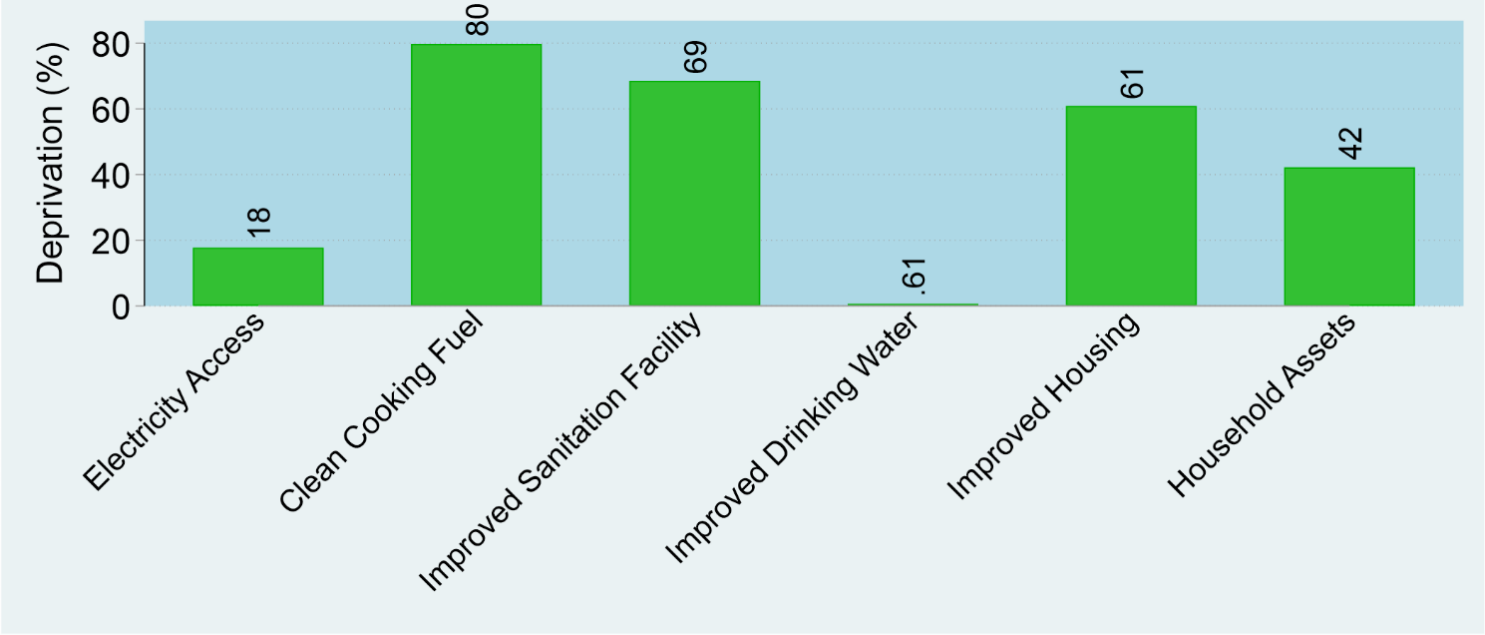
Percentage of deprived women in the selected dimensions of living standard (LS).

The graphical visualization of various indicators of living standard was presented as a deprivation percentage in Fig. 3. It was explained from Fig. 3 that most women living in Bangladesh received the facility of improved drinking water (deprivation in access 0.61%). Approximately, 18% women were found to be deprived in accessing electricity.Use of clean cooking fuels and improved sanitation facility were still very worst in Bangladesh, indicating about 80%, and 69% women deprived in using clean cooking fuels and improved sanitation facility in their households, respectively.

The frequency percentages of several socio-demographic characteristics of women were presented in Table 3. Among the selected women, only one-fourth (26.76%) lived with a high standard of living. More than 50% of women had poor quality of living standard. Approximately 36% of women and their partners were uneducated, 16% of women were educated but their husbands were uneducated, and approximately 38% of women and their husbands were educated. A large percentage of women got married early before reaching the age of 18 (73.6%). More than half (51.7%) were fully empowered to take household decisions, 62.7% had no paid work, 35.6% had 3 or more living children, and about 90% were Muslim. About 20% of women faced wife-beating attitudes by their intimate partners, 63.6% were in rural areas, and about 85% had all-weather road in their community.

**Table 3:** Prevalence of healthcare accessing barriers among the reproductive married women for taking treatment or advice when they sick by background characteristics

		Healthcare Accessing Barriers (%)				
Variables	Frequency Percentage$(\varvec{n}=18,895)$	Getting permission to go for treatment	Getting money for treatment	Distance to health facility	Not wanting to go alone	At least one barrier in accessing healthcare
**Living Standard** ***(p-value)***		***< 0.001***	***< 0.001***	***< 0.001***	***< 0.001***	***< 0.001***
Low	57.59	12.0	52.8	47.5	49.3	73.9
Moderate	15.65	11.0	36.0	37.1	44.8	64.1
High	26.76	7.9	25.3	27.0	34.1	49.8
**Spousal Education** ***(p-value)***		***< 0.001***	***< 0.001***	***< 0.001***	***< 0.001***	***< 0.001***
Both Uneducated	36.3	12.6	58.4	48	49.2	76.5
Wife Educated	16.3	11.6	44.8	42.1	46.4	69.2
Husband Educated	9.8	11.5	45.9	41.2	47.5	68.5
Both Educated	37.6	8.3	26.1	32.1	38.4	53.7
**Women’s Current Age** ***(p-value)***		***< 0.001***	***< 0.001***	***0.013***	***< 0.001***	***< 0.001***
15–20	10.0	13.5	36.0	40.7	53.3	69.0
20–30	36.1	10.7	39.0	38.8	44.0	63.4
30–40	32.0	10.0	45.7	41.3	41.9	65.9
40–49	21.9	10.7	47.9	41.4	45.3	68.8
**Early Marriage** ***(p-value)***		***< 0.001***	***< 0.001***	***< 0.001***	***< 0.001***	***< 0.001***
Yes	73.6	11.3	45.8	42.7	46.4	68.8
No	26.4	9.2	34.5	34	39.4	57.9
**Empowerment** ***(p-value)***		***< 0.001***	***0.001***	***< 0.001***	***< 0.001***	***< 0.001***
Non-empowered	9.6	17.2	39.9	45.0	54.5	71.4
Partially Empowered	38.6	12.9	44.2	43.7	47.2	70.0
Fully Empowered	51.7	7.9	42.3	37.1	40.7	61.9
**Employment Status** ***(p-value)***		***< 0.001***	***< 0.001***	***< 0.001***	***< 0.001***	***< 0.001***
No Paid Work	62.7	11.4	39.1	39.2	44.4	63.7
Agricultural/Unskilled/Domestic Services	23.4	11.0	54.4	47.6	49.6	75.8
Skilled Labor/Sales	8.3	8.1	44.3	35.8	39.1	64.4
Professional/Technical/ Services	5.6	6.8	34.2	30.2	33.1	51.8
**Intimate Partner Violence** ***(p-value)***		***< 0.001***	***< 0.001***	***< 0.001***	***< 0.001***	***< 0.001***
No	80.3	9.8	40.7	38.1	42.6	63.3
Yes	19.7	14.7	51.4	49.7	52.5	76.6
**Religion** ***(p-value)***		***0.350***	***0.028***	***0.015***	***0.008***	***0.052***
Non-Muslim	9.9	10.1	45.2	43	47.4	68
Muslim	90.1	10.8	42.5	40.1	44.2	65.7
**Number of Living Children** ***(p-value)***		***0.489***	***< 0.001***	***< 0.001***	***0.003***	***< 0.001***
≤ 2	64.4	10.6	38.4	38.1	43.7	63.1
> 2	35.6	11.0	50.7	44.5	46.0	71.0
**Place of Residence** ***(p-value)***		***< 0.001***	***< 0.001***	***< 0.001***	***< 0.001***	***< 0.001***
Rural	63.6	12	45.8	45.6	48.3	70.4
Urban	36.4	8.5	37.5	31.2	38	58.2
**Region** ***(p-value)***		***0.001***	***< 0.001***	***0.070***	***0.525***	***< 0.001***
Eastern	25.2	11.9	40.5	40	43.8	62.8
Central	36.4	9.8	44.1	41.5	44.7	66.5
Western	38.4	10.9	43.1	39.6	44.8	67.5
**All-weather Road** ***(p-value)***		***0.803***	***< 0.001***	***< 0.001***	***< 0.001***	***< 0.001***
No	14.9	10.6	51	52.1	50	74.1
Yes	85.1	10.8	41.4	38.3	43.6	64.5

Table 3 also presented the prevalence of healthcare accessing barriers among the reproductive married women for taking treatment or advice by their background characteristics along with p-values. The results of cross-tabulation indicated that improving a woman’s living standard reduces all barriers to accessing healthcare. For example, among women with a lower standard of living, 52.8% had difficulty receiving money for treatment, while only 25.3% of women with high standard of living had this problem. A similar pattern has also been observed in husband-wife education, i.e., educated women faced less barriers if their husbands were also educated, while both groups of educated women who had uneducated husbands and uneducated women who had educated husbands had equal but higher barriers in accessing healthcare (Table 3).

Experiences of barriers to accessing health care also significantly varied by women’s age, early marriage, empowerment status, employment status, intimate partner violence, religion, number of living children, residence type, region, and all-weather road condition. Number of living children and all-weather road did not show significant association with the barrier of permission to go for treatment, and the significant association of region was also not observed with the barrier of not wanting to go alone for treatment. All the selected covariates showing a p < 0.10 at bivariate analysis were considered in the regression models.

### Association of living standard and spousal education with barriers to healthcare access

Table 4 presented the regression results obtained from multiple binary logistic models to examine the adjusted associations of living standard and spousal education with barriers to healthcare access among currently married women in Bangladesh. To check the associations, the important covariates which were significant at bivariate analysis (shown in Table 3) were adjusted in the regression models. The adjusted odds ratio (AOR) of facing barrier in accessing healthcare with 95% confidence intervals (CIs) were calculated by taking the ratio of two odds obtained from two group of women, where odds is defined as the ratio of the probability of facing barrier to the probability of facing no barrier. The adjusted results revealed that increasing the levels of living standard significantly decreases the barriers in accessing healthcare among women. Women with high standard of living had 13% lower odds ((1-0.87)×100 = 13%) of facing barrier in getting permission for treatment (AOR 0.87, 95% CI: 0.76–1.00), 57% lower odds of facing barrier in obtaining money (AOR 0.43, 95% CI: 0.39–0.47), 40% lower odds of facing barrier in distance to health facility (AOR 0.60, 95% CI: 0.55–0.66), 28% lower odds of facing barrier for not wanting to go alone (AOR 0.72, 95% CI: 0.66–0.89), and 44% lower odds of facing at least one barrier to healthcare access (AOR 0.56, 95% CI: 0.52–0.62) in comparison to those with low standard of living. Similar significant pattern of difference was also observed among women having low and moderate standard of living, while the difference of low and moderate standard of living was not existed for the barrier of getting permission to go for treatment.

Spousal education had also significant effect on barriers to healthcare access. Educated women and/or women with educated husbands were less likely to experience barriers to accessing healthcare than educated women whose husbands were also uneducated. For example, women from the educated spouses had 34% lower odds of facing barrier in getting permission for treatment (AOR 0.66, 95% CI: 0.58–0.75), 63% lower odds of facing barrier in getting money (AOR 0.37, 95% CI: 0.34–0.40), 30% lower odds of facing barrier in distance to health facility (AOR 0.70, 95% CI: 0.65–0.76), 25% lower odds of facing barrier in companionship to go for treatment (AOR 0.75, 95% CI: 0.69–0.81), and 51% lower odds of facing at least one barrier to healthcare access (AOR 0.49, 95% CI: 0.45–0.54) compared to those women from uneducated spouses.

The study also found the following variables as potential factors of facing barriers to healthcare access: women’s age, early marriage, empowerment status, employment status, intimate partner violence, religion, number of living children, residence type, region and all-weather road condition (Table 4).

**Table 4:** Odds ratios (OR) with 95% confidence intervals (CI) of healthcare accessing problems obtained from binary logistic regression model after controlling the selected covariates

	OR with 95% CI				
Variables	Getting permission to go for treatment	Getting money for treatment	Distance to health facility	Not wanting to go alone	At least one barrier in accessing healthcare
**Living Standard**					
Low	1.00	1.00	1.00	1.00	1.00
Moderate	1.03 [0.90–1.18]	0.61 [0.56–0.67]***	0.80 [0.73–0.87]***	0.97 [0.89–1.05]	0.80 [0.73–0.88]***
High	0.87 [0.76-1.00]^+^	0.43 [0.39–0.47]***	0.60 [0.55–0.66]***	0.72 [0.66–0.89]***	0.56 [0.52–0.62]***
**Spousal Education**					
Both Uneducated	1.00	1.00	1.00	1.00	1.00
Wife Educated	0.86 [0.74–0.98]*	0.66 [0.60–0.72]***	0.84 [0.77–0.92]***	0.88 [0.80–0.96]**	0.73 [0.66–0.81]***
Husband Educated	0.90 [0.77–1.06]	0.71 [0.64–0.79]***	0.86 [0.77–0.96]**	0.99 [0.89–1.10]	0.76 [0.68–0.86]***
Both Educated	0.66 [0.58–0.75]***	0.37 [0.34–0.40]***	0.70 [0.65–0.76]***	0.75 [0.69–0.81]***	0.49 [0.45–0.54]***
**Women’s Current Age**					
15–20	1.00	1.00	1.00	1.00	1.00
20–30	0.92 [0.79–1.08]	1.14 [1.02–1.28]*	1.04 [0.94–1.17]	0.77 [0.69–0.86]***	0.86 [0.77–0.97]*
30–40	0.85 [0.82-1.00]^+^	1.26 [1.11–1.43]***	1.09 [0.97–1.23]	0.67 [0.60–0.76]***	0.85 [0.75–0.97]*
40–49	0.84 [0.70-1.00]^+^	1.18 [1.02–1.41]*	1.00 [0.87–1.14]	0.71 [0.63–0.81]***	0.84 [0.72–0.96]*
**Early Marriage**					
Yes	1.00	1.00	1.00	1.00	1.00
No	0.82 [0.82–1.04]	0.89 [0.83–0.96]**	0.85 [0.79–0.92]***	0.91 [0.85–0.98]*	0.90 [0.83–0.97]**
**Empowerment Status**					
Non-empowered	1.00	1.00	1.00	1.00	1.00
Partially Empowered	0.76 [0.66–0.88]***	1.13 [1.01–1.26]*	0.95 [0.86–1.06]	0.80 [0.72–0.89]***	0.94 [0.83–1.06]
Fully Empowered	0.46 [0.39–0.53]***	0.96 [0.86–1.08]	0.71 [0.64–0.80]***	0.63 [0.57–0.71]***	0.63 [0.56–0.71]***
**Employment Status**					
No Paid Work	1.00	1.00	1.00	1.00	1.00
Agricultural/Unskilled/ Domestic Services	0.93 [0.83–1.05]	1.30 [1.21–1.41]***	1.14 [1.05–1.23]**	1.12 [1.04–1.20]**	1.34 [1.23–1.46]***
Skilled Manual/Sales	0.78 [0.64–0.95]*	1.24 [1.11–1.39]***	0.91 [0.82–1.02]	0.86 [0.77–0.96]**	1.08 [0.96–1.21]
Professional/Technical/ Services	0.71 [0.56–0.92]**	0.95 [0.83–1.10]	0.87 [0.76-1.00]^+^	0.75 [0.66–0.87]***	0.77 [0.67–0.88]***
**Intimate Partner Violence**					
No	1.00	1.00	1.00	1.00	1.00
Yes	1.40 [1.26–1.56]***	1.25 [1.16–1.35]***	1.39 [1.29–1.50]***	1.34 [1.25–1.45]***	1.56 [1.43–1.70]***
**Religion**					
Non-Muslim		1.00	1.00	1.00	1.00
Muslim		0.80 [0.72–0.89]***	0.80 [0.72–0.88]***	0.80 [0.72–0.88]***	0.79 [0.71–0.88]***
**Number of Living Children**					
≤ 2		1.00	1.00	1.00	1.00
> 2		1.05 [0.97–1.14]	1.04 [0.96–1.13]	1.03 [0.95–1.11]	1.09 [1.00-1.19]*
**Place of Residence**					
Rural	1.00	1.00	1.00	1.00	1.00
Urban	0.81 [0.73–0.91]***	1.21 [1.13–1.31]***	0.77 [0.72–0.83]***	0.85 [0.79–0.91]***	0.93 [0.86-1.00]^+^
**Region**					
Eastern	1.18 [1.04–1.33]	0.89 [0.82–0.97]**	0.93 [0.86-1.00]^+^	0.94 [0.87–1.01]^+^	0.83 [0.77–0.91]***
Central	1.00	1.00	1.00	1.00	1.00
Western	1.10 [0.98–1.23]	0.88 [0.81–0.94]***	0.87 [0.81–0.93]***	0.94 [0.88–1.01]^+^	0.95 [0.88–1.03]
**All-weather Road**					
No		1.00	1.00	1.00	1.00
Yes		0.88 [0.80–0.96]**	0.76 [0.69–0.83]***	2.52 [2.10–3.02]***	0.85 [0.77–0.93]**

*Note*: ^+^p < 0.10; *p < 0.05; **p < 0.01; ***p < 0.001

**Fig. 4:**
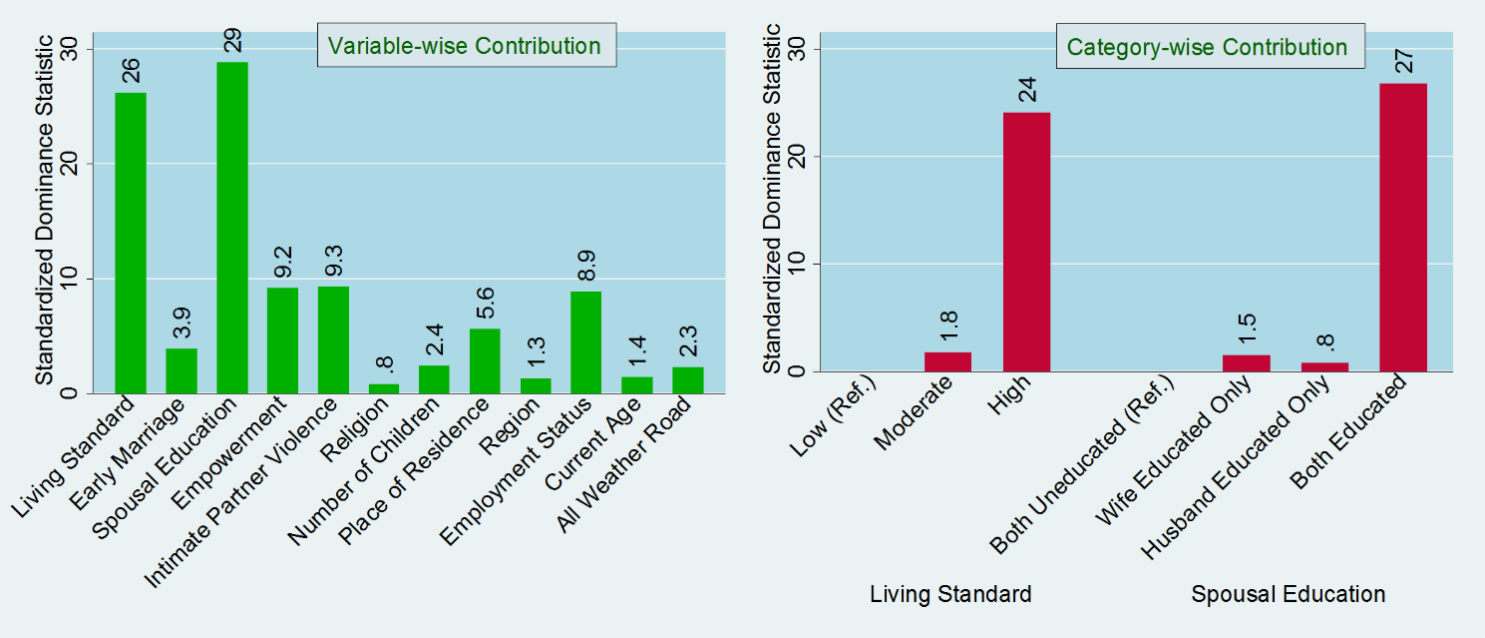
General contribution of the selected predictors including category-wise contribution in the regression model of barriers in accessing healthcare based on the dominance analysis

Figure 4 illustrated the general contribution of the selected predictors to explain the total variation of barriers to healthcare access accounted in the study based on dominance statistics. Among the predictors, living standard and spousal education covered the highest variation in explaining barriers. Living standard contributes to around 26%, whereas spousal education contributes to around 29% to diminish one or more barriers to healthcare access. Again, among the categories of the predictors, high standard of living (24%) and both spousal education (27%) had highest level of contribution in predicting barriers to healthcare access.

## Discussion

In the present study, the barriers to proper health care access for women have been evaluated in terms of getting permission and money to go for treatment, distance to the nearest health facilities, and not wanting to go alone. These factors contribute negatively to ensuring health care in different aspects which leads to the prevalence of poor health outcomes in several health issues [37, 38]. It has been reported in the Supplementary file (see Supplementary Fig. S1 online) that the rate of receiving cares by skilled professional during and after pregnancy is affected due to having more barriers. Moreover, the proportion of non-institutional births, child stunting and low birth weight of children, short birth interval, and underweighted women rises as the number of barriers increase to access proper health care (see Supplementary Fig. S1 online). The findings of the current study provide insights into the associated factors with perceived barriers to receiving proper, efficient, and confidential health care services that women experience in Bangladesh.

The study shows that every two of three women of reproductive age (66%) experience at least one of these four barriers in their lifetime. This percentage is much higher than the neighboring country Myanmar (52%) [5], however, quite similar to some countries having lower-middle-income economies such as South Africa (64.5%) [39], Tanzania (65%) [40], and Rwanda (64%) [20]. However, this finding is slightly low-lying compared to the recent study conducted in Ethiopia (70%) [3].

The present research work investigated the impact of spousal education and living standards on experiencing barrier while accessing healthcare of women and also investigated the magnitude of the impact on having at least one barrier in their lifetime. The living standard was considered as an index which was measured based on six dimensions: electricity access, cooking fuels, sanitation facilities, drinking water, housing materials, and household assets. We followed the equal weighting approach, assigning an equal weight of 1/6 to each dimension. This approach is consistent with the methodology employed in the Human Development Report developed by the Oxford Poverty and Human Development Initiative (OPHI) in collaboration with the United Nations Development Program (UNDP) [30].

In the present study, five different binary logistic regression models had been employed and in all the cases, spousal education and living standard of women played a vital role to significantly influence the health care utilization. Moreover, the study revealed that these two factors together contribute more than 50% control over the accessibility of health seeking behavior of women in Bangladesh. Many studies have been conducted providing strong evidence to justify living conditions as basic social determinants of persons’ physical and mental health outcomes [28, 41]. A recent study showed that women’s life expectancy had been positively influenced by electricity access and female education and negatively associated with women’s adult mortality rate [42]. Thus, experiencing barriers to accessing healthcare plays the role of an intermediate factor that is affected by living standard and hence deteriorates women’s health outcomes. However, there is a lack of relevant studies in Bangladesh to prove the association between living standard and experiencing barriers to women’s health care access. The findings from the study confirm that women maintaining a high and moderate standard of living are less likely to experience at least one barrier to access quality health care compared to those leading a low standard of life. To be specific, getting permission to go for treatment becomes less troublesome for women with a higher standard than women from a lower standard. Moreover, the problem with the issues of getting money for treatment and distance to health facilities elevates as the living standard of women gets worse. However, visiting alone to health centers is considered as a relatively more severe constraint to accessing proper health care for women surviving in a family with moderate to low living standards compared to their counterparts maintaining a high standard of living. However, living standard is considered as a long-run indicator of economic status of a household [43] and also defines the socio-economic status of a region by measuring wealth, material goods, comfort, and available services. It is also claimed that the coverage of the facilities considered as the indicators of living standard such as modern basic sanitation and safe drinking water is the lowest most in the region with poor economy [44, 45]. The financial stability of a household is reflected by its standard of living. A higher standard of living can lead to greater economic stability, which can remove financial barriers to accessing healthcare services among women. When a household has greater financial resources, they are more likely to be able to afford healthcare services and health insurance, which can improve the ability to access healthcare services. Moreover, the cost associated with quality healthcare access gets affordable for women from households with relatively rich economies, and thereby the difficulties with the barriers of obtaining money to ensure proper health care have been reduced for women living their life with a higher standard. Several articles have documented that woman in households with higher economic status are less likely to experience the problem with health access barriers compared to poorer households [5, 7, 46] which is compatible with the result of the study.

Living standard also contributes significantly to the quality of life as it measures wealth, material possessions, and some other factors that define comfort of a household. In the present study, living standard has been measured in terms of access to electricity, modern cooking fuel, sanitation facility, improved drinking water, improved housing materials, and ownership of household assets. Among all these indicators, the percentage of women in Bangladesh who lack clean cooking fuel (80%) is the most, as evident from the study. Globally, about 4 million people are still deprived of clean, safe, reliable, and affordable cooking fuel [47]. This study also proves the importance of the modernization of daily life by ensuring electricity access, transitioning to modern cooking fuel and sanitation facilities, and improving household materials and assets to confirm unimpeded healthcare access for women in Bangladesh.

The study also demonstrated that the barriers associated with the accessibility of quality healthcare are highly influenced by spousal education. It has been evident from the findings that improvement of education level for both partners may reduce the difficulties of healthcare-seeking behavior of women. The odds of experiencing at least one barrier to healthcare for women from households where only husband/ wife or both are educated is less than those where no formal education is observed for both partners. In the case of each particular barrier, lack of education for both partners elevates the risk of perceived barriers to healthcare accessibility. A similar finding was obtained from a study, conducted in Bangladesh, which states that women who are educated and married off to educated partners have better access to healthcare facilities [48]. However, the noticeable fact revealed from the study is that the risk of the problem with the barriers of getting permission to go for treatment and not wanting to go alone cannot be minimized by educating husbands solely. This is ensuring the education of women which is more crucial than their husband’s education to guarantee spontaneous accessibility to healthcare services. Furthermore, in all the cases, women encounter the lowest problem with receiving healthcare access where the spouse are educated. Different studies, conducted in Myanmar [5], Ghana [49], Ethiopia [3], sub-Saharan [10] and East Africa [6], have investigated the impact of education individually for husband and wife and confirmed a negative association between partner’s education and healthcare accessibility barriers which is in agreement with the findings of the study. The plausible reason might be the fact that education of both partners improves the financial ability of a household to afford the expenses associated with healthcare by enhancing higher paid job opportunities [49] and thereby ensures proper healthcare utilization no matter the problem with the issues of distance, getting money, and decision making as well. Moreover, education makes women more aware of their basic human rights and therefore they become more informed of the knowledge of health literacy which in return helps them to overcome the barriers to healthcare accessibility compared to those who have no formal educational attainment [50].

Besides the major findings, the study also reveals some other important factors namely women’s age, early marriage, empowerment status, employment status, intimate partner violence, religion, number of living children, place of residence, region, and all-weather road to have a significant association with quality healthcare access of women. However, the contribution of all these factors together to healthcare access is lower compared to the combined contribution of the factors spousal education and living standard of women. This is because higher living standard and educational attainment uphold a woman’s socio-economic status which makes them independent and thereby enable them to overcome all sorts of decision-making barriers to ensure unhindered healthcare access.

The study has some limitations. Only 4 items were considered in the study that can create bias in defining healthcare accessing barriers. Some other important items like ‘knowing where to go to seek care’, ‘having to take transportation’ and ‘concern that there may not be a female health provider’ are also important to get actual scenario of healthcare accessing barriers which were not available in the BDHS, 2017-18. Again, as the cross-sectional data has been used in the study, it is difficult to show the causal relationship of education and living standard with health access barriers. The results of this study cannot be generalized to all women in Bangladesh because the study did not consider the following women: divorced, separated or not living together with husband. Furthermore, the living standard is not an absolute measure. It was measured using six dimensions taking equal weights, where all the indicators may not be equally important. Therefore, further study can improve the measure of living standard taking with unequal weights or adding more dimensions.

## Conclusion and policy implications

In Bangladesh, about two-thirds of reproductive women face barriers to accessing healthcare. To break down the barriers, the current study provides sufficient evidence to prove that standard of living and spousal education are the relevant factors that have a profound effect on women’s health-seeking hurdles. The likelihood of experiencing barriers for the women maintaining a higher standard of living, who are educated and got married to educated partners is the lowermost. In order to facilitate access to quality healthcare and progressive health outcomes for women and newborns, it is essential to alleviate perceived barriers to accessing healthcare. Therefore, to attain universal health-access coverage by ignoring the obstacles to accessing healthcare, the findings suggest decentralizing the healthcare service and taking initiatives to promote health education to the region with moderate to low living standards. Policies should also target strengthening women and their partners by developing interventions that can outreach equity-based educational coverage to overcome the propensity of the barriers to accessing healthcare amenities. Governments can implement policies to address gender inequality for healthcare access, such as promoting women’s empowerment, protecting women’s rights, and increasing women’s representation in leadership positions. Moreover, government-private partnership can promote health insurance coverage, particularly for women having low standard of living, by providing subsidies or tax incentives for purchasing health insurance. The present study believes that reinforcing the prevailing strategies according to the findings of the study to alleviate hindered access to healthcare services can contribute significantly to achieving SDGs 3.1, 3.7, and 3.8.

## Electronic supplementary material

Below is the link to the electronic supplementary material.

Supplementary Material 1

## Data Availability

The Bangladesh Demographic and Health Survey (BDHS), 2017-18 data and relevant materials used in this study are publicly available at https://dhsprogram.com/data/dataset/Bangladesh_Standard-DHS_2017.cfm?flag=0.

## Abbreviations

ANC: Antenatal care
BDHS: Bangladesh Demographic and Health Survey
DC: Delivery care
EA: LS:living standard enumeration area
MMR: Maternal mortality ratio
OPHI: Oxford Poverty and Human Development Initiative
PNC: Postnatal care
PPS: Probability proportional to size
UHC: Universal health coverage
UNDP: United Nations Development Program
SDG: Sustainable development goal

## References

[1] Bauer MS, Williford WO, McBride L, McBride K, Shea NM (2005). Perceived barriers to health care access in a treated population. Int J Psychiatry Med.

[2] UNEP and IEA. Energy Subsidy Reform and Sustainable Development: Challenges for policymakers. Dep Econ Soc Aff. 2001;:1–27.

[3] Tamirat KS, Tessema ZT, Kebede FB (2020). Factors associated with the perceived barriers of health care access among reproductive-age women in Ethiopia: a secondary data analysis of 2016 ethiopian demographic and health survey. BMC Health Serv Res.

[4] Zegeye B, El-Khatib Z, Ameyaw EK, Seidu AA, Ahinkorah BO, Keetile M (2021). Breaking barriers to healthcare access: a multilevel analysis of individual- and community-level factors affecting women’s access to healthcare services in Benin. Int J Environ Res Public Health.

[5] Htun NMM, Hnin ZL, Khaing W (2021). Empowerment and health care access barriers among currently married women in Myanmar. BMC Public Health.

[6] Minyihun A, Tessema ZT (2020). Determinants of access to health care among women in east african countries: a multilevel analysis of recent demographic and health surveys from 2008 to 2017. Risk Manag Healthc Policy.

[7] Seidu AA, Agbaglo E, Dadzie LK, Ahinkorah BO, Ameyaw EK, Tetteh JK (2021). Individual and contextual factors associated with barriers to accessing healthcare among women in Papua New Guinea: insights from a nationwide demographic and health survey. Int Health.

[8] WHO (2019). Maternal mortality.

[9] Say L, Chou D, Gemmill A, Tunçalp Ö, Moller AB, Daniels J (2014). Global causes of maternal death: a WHO systematic analysis. Lancet Glob Heal.

[10] Seidu AA (2020). Mixed effects analysis of factors associated with barriers to accessing healthcare among women in sub-saharan Africa: insights from demographic and health surveys. PLoS ONE.

[11] BDHS. National Institute of Population Research and Training (NIPORT), and ICF (2020). Bangladesh Demographic and Health Survey 2017-18. Dhaka, Bangladesh, and Rockville.

[12] Ahinkorah BO, Budu E, Seidu AA, Agbaglo E, Adu C, Ameyaw EK (2021). Barriers to healthcare access and healthcare seeking for childhood illnesses among childbearing women in sub-saharan Africa: a multilevel modelling of demographic and health surveys. PLoS ONE.

[13] Habibov N, Fan L. The effect of maternal healthcare on the probability of child survival in Azerbaijan. Biomed Res Int. 2014;2014. 10.1155/2014/317052 PMC4119731 25110673

[14] Lassi ZS, Majeed A, Rashid S, Yakoob MY, Bhutta ZA (2013). The interconnections between maternal and newborn health-evidence and implications for policy. J Matern Neonatal Med.

[15] Limenih MA, Endale ZM, Dachew BA (2016). Postnatal Care Service utilization and Associated factors among women who gave birth in the last 12 months prior to the study in Debre Markos Town, Northwestern Ethiopia: A Community-Based cross-sectional study. Int J Reprod Med.

[16] Pulok MH, Sabah MNU, Uddin J, Enemark U. Progress in the utilization of antenatal and delivery care services in Bangladesh: where does the equity gap lie? BMC Pregnancy Childbirth. 2016;16. 10.1186/s12884-016-0970-4 PMC4967314 27473150

[17] Silumbwe A, Nkole T, Munakampe MN, Milford C, Cordero JP, Kriel Y (2018). Community and health systems barriers and enablers to family planning and contraceptive services provision and use in Kabwe District, Zambia. BMC Health Serv Res.

[18] Kohan S, Simbar M, Taleghani F (2012). Women’s experience regarding the role of health centers in empowering them for family planning. Iran J Nurs Midwifery Res.

[19] George S, Daniels K, Fioratou E (2018). A qualitative study into the perceived barriers of accessing healthcare among a vulnerable population involved with a community centre in Romania. Int J Equity Health.

[20] Nisingizwe MP, Tuyisenge G, Hategeka C, Karim ME (2020). Are perceived barriers to accessing health care associated with inadequate antenatal care visits among women of reproductive age in Rwanda?. BMC Pregnancy Childbirth.

[21] Hossen A, Westhues A (2011). Improving access to government health care in rural Bangladesh: the voice of older adult women. Health Care Women Int.

[22] Mohiuddin AK (2020). An extensive review of patient satisfaction with healthcare services in Bangladesh. Patient Exp J.

[23] Directorate General of Health Services (DGHS). Directorate General of Family Planning (DGFP). Bangladesh National Strategy for maternal Health 2019–2030. Gov People’s Repub Bangladesh Minist Heal Fam Welf; 2019.

[24] Chowdhury AMR, Perry HB (2020). NGO Contributions to Community Health and Primary Health Care: Case Studies on BRAC (Bangladesh) and the Comprehensive Rural Health Project, Jamkhed (India). Oxf Res Encycl Glob Public Heal.

[25] Hahn RA, Truman BI (2015). Education improves public health and promotes health equity. Int J Heal Serv.

[26] Bhat RA (2015). Role of education in the empowerment of women in India. J Educ Pract.

[27] Joseph J. Perspectives on gender inequality and the barrier of culture on education. J Community Posit Pract. 2012;:769–89.

[28] Gu J, Ming X. The influence of living conditions on self-rated health: evidence from china. Int J Environ Res Public Health. 2021;18. 10.3390/ijerph18179200 PMC8431523 34501800

[29] Parsaei R, Roohafza H, Feizi A, Sadeghi M, Sarrafzadegan N (2020). How different stressors affect quality of life: an application of multilevel latent class analysis on a large sample of industrial employees. Risk Manag Healthc Policy.

[30] Alkire S, Foster J (2011). Counting and multidimensional poverty measurement. J Public Econ.

[31] Islam UN, Sen KK, Bari W (2022). Living standard and access to tetanus toxoid immunization among women in Bangladesh. BMC Public Health.

[32] Rahman M, Curtis SL, Chakraborty N, Jamil K (2017). Women’s television watching and reproductive health behavior in Bangladesh. SSM - Popul Heal.

[33] Karmaker SC, Sen KK, Singha B, Hosan S, Chapman AJ, Saha BB (2022). The mediating effect of energy poverty on child development: empirical evidence from energy poor countries. Energy.

[34] Webb NJ, Miller TL, Stockbridge EL (2022). Potential effects of adverse childhood experiences on school engagement in youth: a dominance analysis. BMC Public Health.

[35] Tonidandel S, LeBreton JM (2010). Determining the relative importance of predictors in logistic regression: an extension of relative weight analysis. Organ Res Methods.

[36] Stadler M, Cooper-Thomas HD, Greiff S (2017). A primer on relative importance analysis: illustrations of its utility for psychological research. Psychol Test Assess Model.

[37] Bohren MA, Hunter EC, Munthe-Kaas HM, Souza JP, Vogel JP, Gülmezoglu AM (2014). Facilitators and barriers to facility-based delivery in low- and middle-income countries: a qualitative evidence synthesis. Reprod Health.

[38] Moyer CA, Mustafa A. Drivers and deterrents of facility delivery in sub-saharan Africa: a systematic review. Reprod Health. 2013;10. 10.1186/1742-4755-10-40 PMC3751820 23962135

[39] Silal SP, Penn-Kekana L, Harris B, Birch S, McIntyre D. Exploring inequalities in access to and use of maternal health services in South Africa. BMC Health Serv Res. 2012;12. 10.1186/1472-6963-12-120 PMC3467180 22613037

[40] Bintabara D, Nakamura K, Seino K (2018). Improving access to healthcare for women in Tanzania by addressing socioeconomic determinants and health insurance: a population-based cross-sectional survey. BMJ Open.

[41] Barskova T, Oesterreich R (2009). Post-traumatic growth in people living with a serious medical condition and its relations to physical and mental health: a systematic review. Disabil Rehabil.

[42] Rahman MM, Alam K (2021). The role of access to electricity, female education, and public health expenditure on female health outcomes: evidence from SAARC-ASEAN countries. BMC Womens Health.

[43] Heath R (2014). Women’s Access to Labor Market Opportunities, Control of Household Resources, and domestic violence: evidence from Bangladesh. World Dev.

[44] UNICEF, WHO (2011). Drinking water: equity, Safety and sustainability.

[45] UNICEF, WHO (2012). Progress on drinking Water and Sanitation: 2012 update.

[46] Pons-Duran C, Lucas A, Narayan A, Dabalen A, Menéndez C (2019). Inequalities in sub-saharan african women’s and girls’ health opportunities and outcomes: evidence from the demographic and health surveys. J Glob Health.

[47] Energy Sector Management Assistance Program (2020). The state of Access to Modern Energy Cooking Services.

[48] Hasan MN, Uddin MSG (2016). Women empowerment through health seeking behavior in Bangladesh: evidence from a national survey. South East Asia J Public Heal.

[49] Seidu AA, Darteh EKM, Agbaglo E, Dadzie LK, Ahinkorah BO, Ameyaw EK (2020). Barriers to accessing healthcare among women in Ghana: a multilevel modelling. BMC Public Health.

[50] Berkman ND, Sheridan SL, Donahue KE, Halpern DJ, Crotty K (2011). Low health literacy and health outcomes: an updated systematic review. Ann Intern Med.

