# Supplementary material for: Do education and living standard matter in breaking barriers to healthcare access among women in Bangladesh?
Source: BMC Public Health. 2023 Jul 26;23:1431. doi: 10.1186/s12889-023-16346-8 (PMC10373322; doi:10.1186/s12889-023-16346-8)
Supplement: Supplementary file 1 — Supplementary Material 1 [file 12889_2023_16346_MOESM1_ESM.docx]

Supplementary Fig. S1. Proportion of receiving healthcare from skilled health attendants during antenatal care (ANC), delivery and postnatal care (PNC), low birth weight of newborn babies, child stunting, underweight of women, non-institutional deliveries and short birth interval (below the interval of 33 months between two consecutive live births) with respect to barriers to healthcare access among women in Bangladesh obtained from analyzing BDHS, 2017-18 data.
